# Supplementary material for: Neuroprotective and Anti-Inflammatory Effects of Dimethyl Fumarate, Monomethyl Fumarate, and Cannabidiol in Neurons and Microglia
Source: Int J Mol Sci. 2024 Dec 5;25(23):13082. doi: 10.3390/ijms252313082 (PMC11642486; doi:10.3390/ijms252313082)
Supplement: Supplementary file 1 [file ijms-25-13082-s001.zip › Supplementary Figures Footnotes.pdf]

Supplementary Figure 1. Representative immunofluorescence images from microglia showing NF-kB p65 protein location (green). TO-PRO (blue) was used as a nuclear counterstain. Cells were treated for 30 minutes with vehicle (VEH), lipopolysaccharide (LPS), dimethyl fumarate (DMF) 30 µM, monomethyl fumarate (MMF) 30 µM or cannabidiol (CBD) 6 µM. Scale bar: 20 µm, shown in the bottom-right corner of each image.

Supplementary Figure 2. Representative immunofluorescence images from apoptosis in neurons. Neurons were pre-treated for 4 hours with dimethyl fumarate (DMF) 10 µM, monomethyl fumarate (MMF) 10 µM or cannabidiol (CBD) 7 µM. Then, the supernatants of 48-hour lipopolysaccharide (LPS)-treated or LPS + drug (at the same drug concentrations as in neurons) microglia cells were added to the neuronal culture and incubated for an additional 4 hours. Apoptosis was determined using the TUNEL assay. Positive apoptotic cells are marked in green. All images were captured under identical exposure and imaging settings to ensure consistency across all experimental groups. Scale bar: 50 µm, shown in the bottom-right corner of each image.

Supplementary Figure 3. Principal component analysis of the gene expression profile of neurons treated with DMF, MMF or CBD. A. Neurons treated with DMF 30 µM or vehicle (DMSO) for 4 and 24 hours. **B.** Neurons treated with MMF 30 µM or vehicle (DMSO) for 4 and 24 hours. **C.** Neurons treated with CBD 6 µM or vehicle (EtOH) for 4 and 24 hours. **A-C.** Each condition presents experimental triplicates.

Supplementary Figure 4. Differentially expressed genes (DEGs) in neurons treated with DMF, MMF or CBD compared to vehicle. A. Volcano plot of the DEGs in neurons treated with DMF 30

µM (4h) compared to DMSO. **B.** Volcano plot of the DEGs in neurons treated with DMF 30 µM (24h) compared to DMSO. **C.** Volcano plot of the DEGs in neurons treated with MMF 30 µM (4h) compared to DMSO. **D.** Volcano plot of the DEGs in neurons treated with MMF 30 µM (24h) compared to DMSO. **E.** Volcano plot of the DEGs in neurons treated with CBD 6 µM (4h) compared to EtOH. **F.** Volcano plot of the DEGs in neurons treated with CBD 6 µM (24h) compared to EtOH. **A-F.** DEGs with adjusted p-value<0.05 and fold changes>0.5 or <-0.5 are represented. Red plots represent upregulated genes; blue plots represent downregulated genes; grey plots represent genes with no significant differences or smaller fold changes. The 25 most relevant DEGs (using the Manhattan distance) are indicated with their name. The x-axis represents the fold change of the gene expression between drug-treated cells compared to their vehicle. The y-axis represents the -log10 of the adjusted p-value.

Supplementary Figure 5. Fold change of the differentially expressed genes (DEGs) in neurons treated with DMF, MMF or CBD. A. Neurons treated with DMF 30 µM (4h) compared to DMSO. **B.** Neurons treated with DMF 30 µM (24h) compared to DMSO. **C.** Neurons treated with MMF 30 µM (4h) compared to DMSO. **D.** Neurons treated with MMF 30 µM (24h) compared to DMSO. **E.** Neurons treated with CBD 6 µM (4h) compared to EtOH. **F.** neurons treated with CBD 6 µM (24h) compared to EtOH. **A-F.** The x-axis represents the number of DEGs and the y-axis represents the log2 of the fold change of the gene expression between drug and vehicle. All the genes with a p-adj<0.05 are represented.

Supplementary Figure 6. Transcriptomic changes induced by LPS in microglia cells. A. Principal component analysis of the gene expression profile of microglia cells treated with LPS or vehicle (DMSO or EtOH) for 4 and 24 hours. Each condition presents experimental triplicates. **B.** Venn diagram showing the number and overlap of the differentially expressed genes (DEGs) in

microglia cells treated with LPS for 4 and 24 hours compared to vehicles. **C.** Volcano plot of the DEGs in microglia treated with LPS (4h) compared to DMSO. **D.** Volcano plot of the DEGs in microglia treated with LPS (24h) compared to DMSO. **E.** Volcano plot of the DEGs in microglia treated with LPS (4h) compared to EtOH. **F.** Volcano plot of the DEGs in microglia treated with LPS (24h) compared to EtOH. **C-F.** DEGs with adjusted p-value<0.05 and fold changes>0.5 or <-0.5 are represented. Red plots represent upregulated genes; blue plots represent downregulated genes; grey plots represent genes with no significant differences or smaller fold changes. The 25 most relevant DEGs (using the Manhattan distance) are indicated with their name. The x-axis represents the fold change of the gene expression between drug-treated cells compared to their vehicle. The y-axis represents the -log10 of the adjusted p-value.

Supplementary Figure 7. Common differentially expressed genes (DEGs) by LPS treatment between vehicles. A. Venn diagram showing the number and overlap of the DEGs induced by 4 hours of LPS treatment compared to DMSO or EtOH. **B.** Venn diagram showing the number and overlap of the DEGs induced by 24 hours of LPS treatment compared to DMSO or EtOH. **C.** Venn diagram showing the number and overlap between 4 and 25 hours of the common DEGs between vehicles. **A-C.** DEGs are separated according to their up or downregulation. The percentage of common DEGs is indicated for each condition.

Supplementary Figure 8. Differentially expressed genes (DEGs) in LPS-activated microglia cells treated with DMF, MMF or CBD. A. Volcano plot of the DEGs in microglia treated with DMF 30 µM (4h) compared to LPS. **B.** Volcano plot of the DEGs in microglia treated with DMF 30 µM (24h) compared to LPS. **C.** Volcano plot of the DEGs in microglia treated with MMF 30 µM (4h) compared to LPS. **D.** Volcano plot of the DEGs in microglia treated with MMF 30 µM (24h) compared to LPS. **E.** Volcano plot of the DEGs in microglia treated with CBD 6 µM (4h) compared

to LPS. **F.** Volcano plot of the DEGs in microglia treated with CBD 6 µM (24h) compared to LPS. **A-F.** DEGs with adjusted p-value<0.05 and fold changes>0.5 or <-0.5 are represented. Red plots represent upregulated genes; blue plots represent downregulated genes; grey plots represent genes with no significant differences or smaller fold changes. The 25 most relevant DEGs (using the Manhattan distance) are indicated with their name. The x-axis represents the fold change of the gene expression between drug-treated cells compared to their vehicle. The y-axis represents the -log10 of the adjusted p-value.

Supplementary Figure 9. Fold change of the differentially expressed genes (DEGs) in microglia cells treated with DMF, MMF or CBD. A. Microglia cells treated with LPS + DMF 30 µM (4h) compared to LPS. **B.** Microglia cells treated with LPS + DMF 30 µM (24h) compared to LPS. **C.** Microglia cells treated with LPS + MMF 30 µM (4h) compared to LPS. **D.** Microglia cells treated with LPS + MMF 30 µM (24h) compared to LPS. **E.** Microglia cells treated with CBD 6 µM (4h) compared to LPS. **F.** Microglia cells treated with CBD 6 µM (24h) compared to LPS. **A-F.** The x-axis represents the number of DEGs and the y-axis represents the log2 of the fold change of the gene expression between drug and LPS. All the genes with a p-adj<0.05 are represented.
