# Supplementary figures and images for: Neuroprotective and Anti-Inflammatory Effects of Dimethyl Fumarate, Monomethyl Fumarate, and Cannabidiol in Neurons and Microglia
Source: Int J Mol Sci. 2024 Dec 5;25(23):13082. doi: 10.3390/ijms252313082 (PMC11642486; doi:10.3390/ijms252313082)

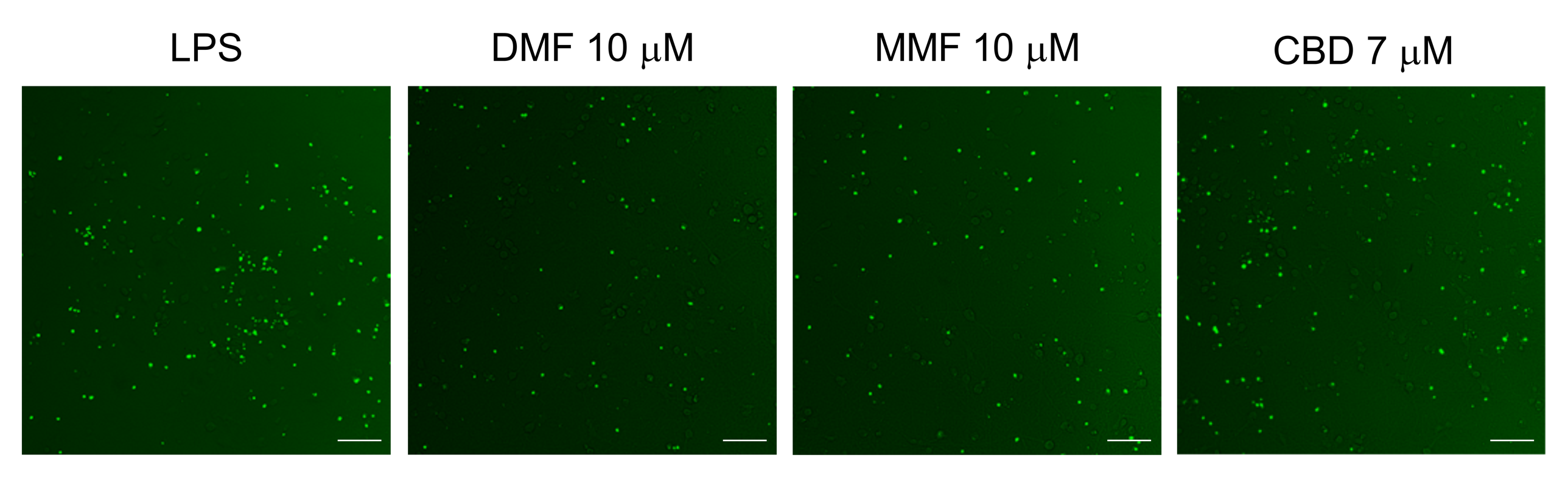

Supplement: Supplementary file 1 [file ijms-25-13082-s001.zip › SUPPLEMENTARY FIGURE 2.tif]

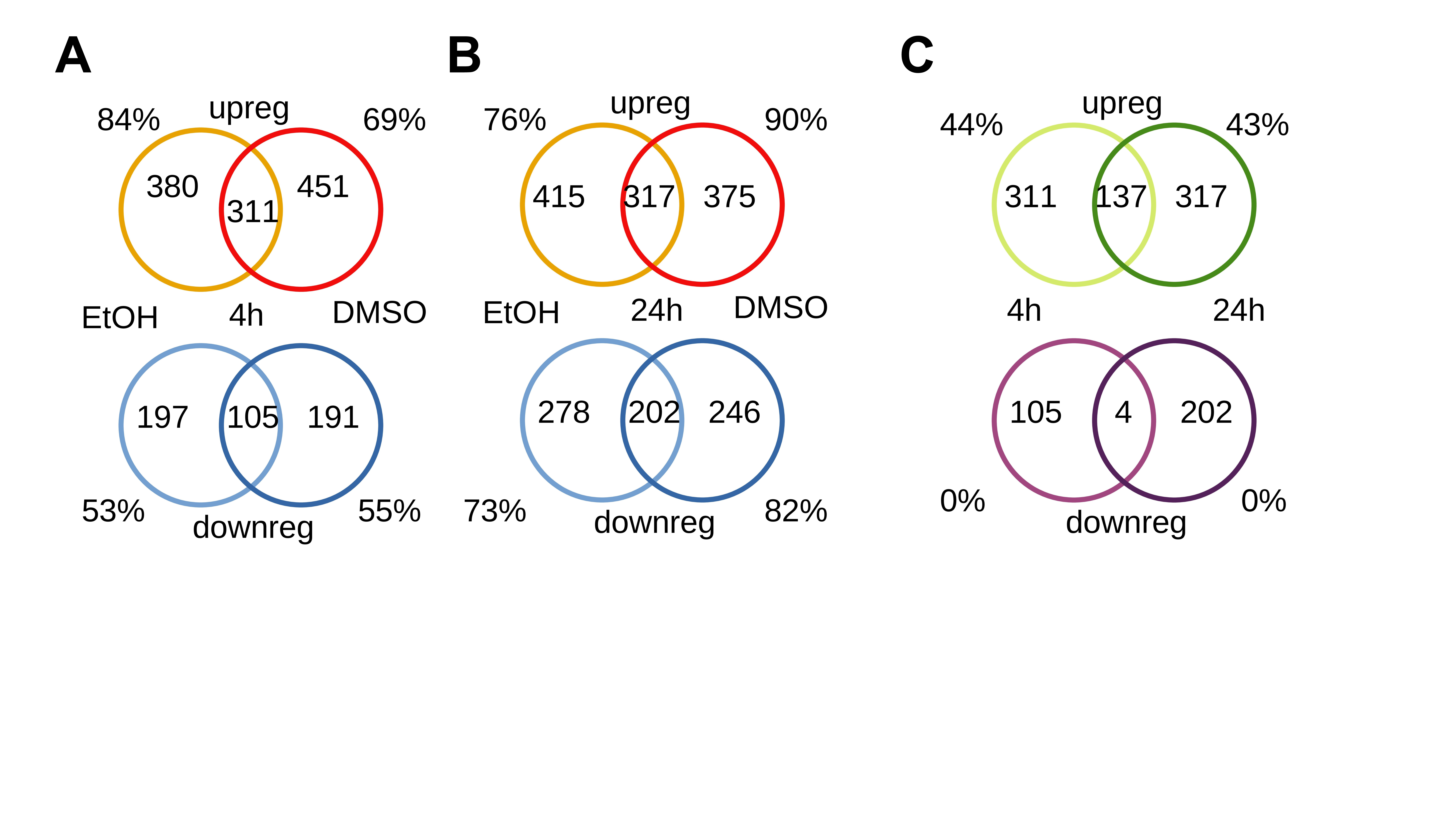

Supplement: Supplementary file 1 [file ijms-25-13082-s001.zip › SUPPLEMENTARY FIGURE 7.tif]
